# Software to manage regulatory workflows for medical device development at academic medical centers: A critical gap

**DOI:** 10.1017/cts.2023.2

**Published:** 2023-02-15

**Authors:** Lynn Rose, Jennifer McCaney, Gaurav Dave, Kimberly A. Brownley, David Sheridan, Payal Shah, Grzegorz Zapotoczny, Juan Espinoza

**Affiliations:** 1 Department of Pharmacy, University of Washington, Seattle, WA, USA; 2 Anderson School of Management, University of California Los Angeles, Los Angeles, CA, USA; 3 Department of Medicine, The University of North Carolina at Chapel Hill, Chapel Hill, NC, USA; 4 North Carolina Translational and Clinical Sciences Institute, The University of North Carolina at Chapel Hill, Chapel Hill, NC, USA; 5 Center for Health Equity Research, The University of North Carolina at Chapel Hill, Chapel Hill, NC, USA; 6 Department of Psychiatry, The University of North Carolina at Chapel Hill, Chapel Hill, NC, USA; 7 Department of Emergency Medicine, Oregon Health & Science University, Portland, OR, USA; 8 West Coast Consortium for Technology & Innovation in Pediatrics, Children’s Hospital Los Angeles, Los Angeles, CA, USA; 9 Division of General Pediatrics, Children’s Hospital Los Angeles, Los Angeles, CA, USA; 10 Department of Pediatrics, Keck School of Medicine of USC, Los Angeles, CA, USA

**Keywords:** Medical device development, research infrastructure, regulatory requirements, software, artificial intelligence

## Introduction

Research from academic institutions is a significant driving force in developing new medical devices. In 2020, the Association of University Technology Managers (AUTM) reported 27,112 invention disclosures [1]. Despite these impressive efforts, disclosures do not translate into commercialized products due to numerous barriers, including weak support infrastructure and the lack of institutional knowledge in regulatory sciences. There is a critical need to develop and deploy tools and processes that support regulatory management in academic settings.

## Critical Gaps

Medical device development is a complex and highly regulated process that can take 3–10 years and $31–94 M of investment to reach commercialization [2]. Regulatory preparedness plays a critical role in clinical trial activation as over half of early-stage medical device development activities are associated with regulatory processes [3]. Most universities have limited regulatory expertise, and university technology transfer offices are resource-limited in how far and to what degree they can assist innovators in keeping abreast of regulatory guidance and decision-making. Barriers repeatedly identified by academic investigators include 1) limited understanding of the regulatory requirements for design controls and market approval [4]; 2) a lack of funding to cover the high cost of product design and development [5], and 3) the changing environment of information technology and security [6].

The sheer number of relevant guidance documents, testing protocols, and submission checklists for translational researchers to follow present a significant barrier to catalyzing translational efficiency and commercialization. From the onset, researchers have to make critical decisions regarding their biomedical device(s), such as materials selection, benchtop testing, animal models, and clinical study protocols which, if incomplete or inadequately documented, can undermine an otherwise successful premarket application. The consequences of potential missteps are evident in the US Food and Drug Administration (FDA) Center for Devices and Radiological Health (CDRH) as one-third of applications are rejected before the review due to errors and refuse-to-accept (RTA) rates for applications are high. The 510(k) pathway, the most common pathway for medical technology, representing over 3000 applications in 2020, exhibited an average RTA rate of 32.43% [7]. Within certain CDRH Offices, the average RTA is as high as 46% with rejection rates over 60% [7]. Common reasons for an RTA include but are not limited to (1) inaccurate device description, (2) incorrect predicate identification for the determination of substantial equivalence, (3) incorrect indication for use or labeling, (4) lack of sterilization or reprocessing information, (5) lack of biocompatibility testing or incomplete materials identification, and (6) irrelevant or inadequate test data. Currently, resources within the academic research institutions are not positioned to address the scale and complexity of the rapidly evolving regulatory requirements. Studies on the general dissemination of regulatory and research information show that the standard approach to disseminating information, e.g., by publication, is ineffective. Information uptake does not occur spontaneously nor in a product-specific manner. When it does, it is often not standardized across diverse settings leading to ineffective implementation [8]. Furthermore, there is a need to streamline the regulatory and FDA submission workflows to manage a widening range of medical technologies.

The FDA and the National Institutes of Health (NIH) have taken steps to help alleviate some of these challenges for medical device developers by creating or funding a number of programs at academic institutions (Table 1). A common feature of these programs is individualized support from advisory committees on medical, engineering, regulatory, and marketing processes. These committees comprise individuals with extensive experience in the MedTech industry and provide valuable feedback based on investigator progress reports [9,10]. However, these programs are difficult to scale due to their reliance on limited numbers of experts. Software-based solutions have been successfully deployed in many aspects of academic research infrastructure to streamline, standardize, and multiply research efforts. Applications like REDCap for electronic data capture, OnCore for Clinical trials management, and i2b2 for cohort discovery have been widely adopted by academic medical centers. Unfortunately, there are few software options that support the needs of medical device developers Tools like Greenlight Guru for quality management and Nyquist Data for predicate discovery were developed for industry and have limited applicability and uptake in an academic setting due to their pricing and the need for personalized “translation” of the regulatory terms and process beforehand.

**Table 1.** National and institutional programs with federal funding support that address medical device development

Program	Link	Support
NIH Clinical and Translational Science Award (CTSA) Regulatory Knowledge and Support (RKS) cores	https://ncats.nih.gov/ctsa	NIH
Pediatric Device Consortia	https://www.fda.gov/industry/medical-products-rare-diseases-and-conditions/pediatric-device-consortia-grants-program	FDA
I-Corps	https://beta.nsf.gov/funding/initiatives/i-corps	NSF
The West Coast Consortium for Technology & Innovation in Pediatrics (CTIP)	https://www.westcoastctip.org/	FDA
University of Washington (UW) WE-REACH program	https://www.washington.edu/we-reach/	NIH
Oregon Health Sciences University (OHSU) Biomedical Innovation Program	https://www.ohsu.edu/octri/biomedical-innovation-program-academia-marketplace	NIH
University of California Los Angeles (UCLA) Biodesign Program	https://biodesign.ucla.edu/	Other
University of North Carolina Regulatory Guidance for Academic Research of Drugs and Devices (ReGARDD) program	https://www.regardd.org/	NIH
Center for the Translation of Rehabilitation Engineering Advances and Technology (TREAT)	https://www.treatcenter.org/	NIH
Stanford Byers Center for Biodesign	https://biodesign.stanford.edu/	Other

FDA, Food and Drug Administration; NIH, National Institutes of Health; NSF, National Science Foundation.

In response to the aforementioned challenges, we see a significant need to develop better artificial intelligence (AI)-driven software solutions that will enable academic medical centers to scale medical device management in a cost-effective, sustainable, and accessible way. AI-driven software can reduce reliance on regulatory experts for many early tasks, including product risk classification (Classes I–III); product code selection; identifying applicable federal regulations (e.g. special controls); suggesting predicate devices; recommending testing protocols; and surfacing relevant guidance documents. A regulatory support engine that uses optical character recognition can potentially extract relevant information using text mining and natural language processing from existing FDA, EUDAMED, clinical trials, regulatory guidance, 510(k) summaries, or PubMed databases, as well as from ISO-recognized standards.

However, software alone is rarely the solution. All technologies require the appropriate people and processes in place to generate value. For example, simply installing a REDCap instance will not solve an institution’s data collection difficulties. Project managers, support teams, training sessions, office hours, and more are necessary to meaningfully derive value from REDCap. Regulatory management software will be no different. Today, almost nothing is known about how best to deploy this software at academic medical centers. It will be critical to develop and evaluate programmatic solutions that address the implementation, evaluation, and maintenance of these tools.

## Conclusion

Our experiences as regulatory consultants in academic medical centers have identified a critical need for scalable tools that would accurately guide academic medical device inventors through the regulatory process and testing requirements for their devices. The medical device management program we envision would leverage AI-driven software to enable early assessment of the regulatory classification and requirements for new medical devices, while providing guidance and support to investigators. This approach will lower the barriers to entry, level the playing field for researchers, and accelerate the pace of medical device development at academic institutions.
